# Supplementary material for: AP-4-mediated axonal transport controls endocannabinoid production in neurons
Source: Nat Commun. 2022 Feb 25;13:1058. doi: 10.1038/s41467-022-28609-w (PMC8881493; doi:10.1038/s41467-022-28609-w)
Supplement: Supplementary file 1 — Supplementary Information [file 41467_2022_28609_MOESM1_ESM.pdf]

AP-4-mediated axonal transport controls endocannabinoid production in neurons

Alexandra K. Davies, Julian E. Alecu, Marvin Ziegler, Catherine G. Vasilopoulou, Fabrizio Merciai, Hellen Jumo, Wardiya Afshar-Saber, Mustafa Sahin, Darius Ebrahimi-Fakhari, Georg H. H. Borner

Nature Communications

Supplementary Information

Supplementary Figures 1-8

Supplementary Table 1

Supplementary References

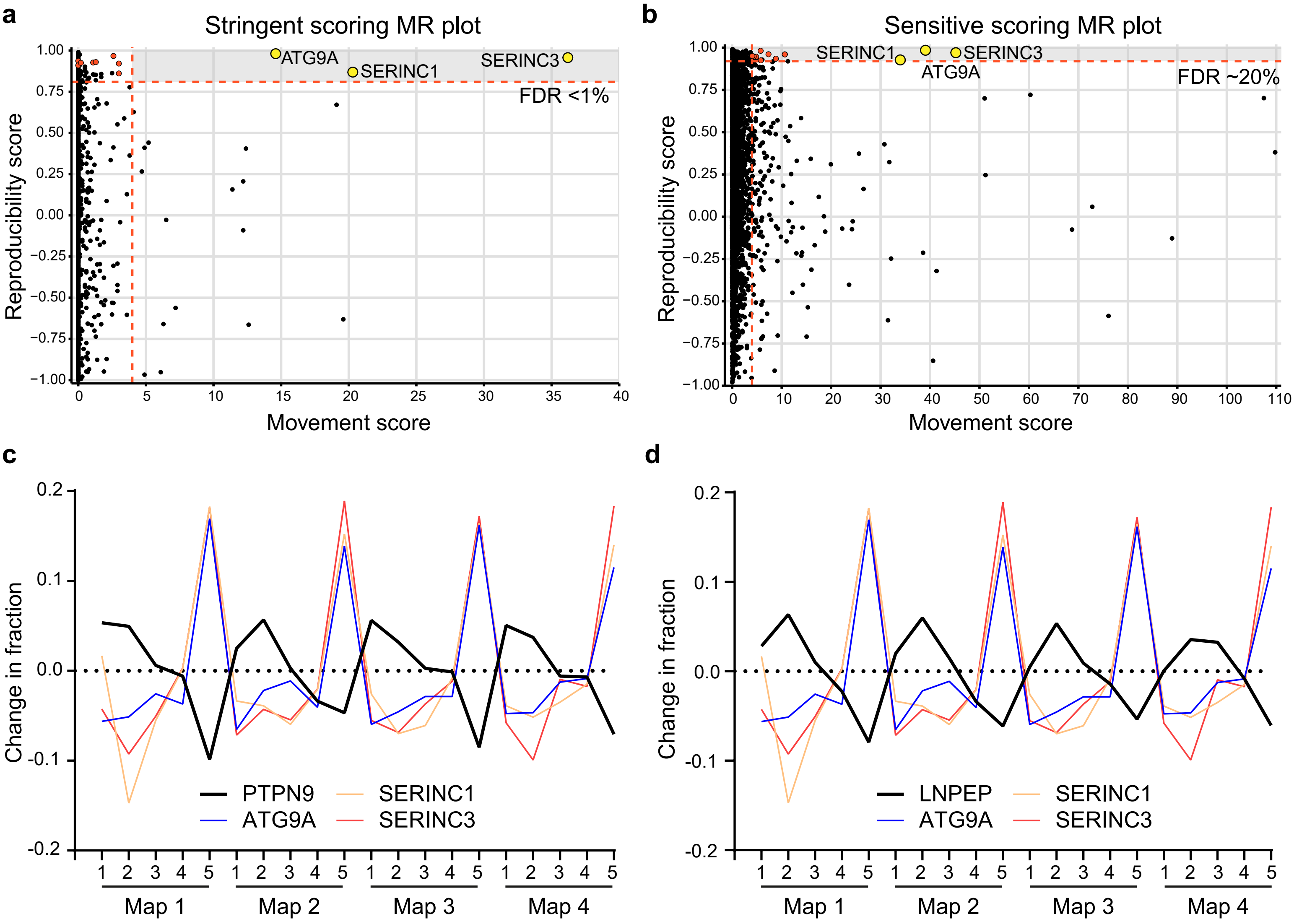

Supplementary Figure 1. Sensitive analysis of Dynamic Organellar Maps identifies DAGLB as an AP-4 cargo protein. Dynamic Organellar Maps of *AP4B1* knockout (KO) and *AP4E1* KO HeLa cells were compared to maps of wild-type HeLa cells (each in duplicate, totalling 4 comparisons). For 3926 proteins profiled across all maps, the 'MR' plots display the magnitude of shift (M) and the within-clone reproducibility of shift direction (R). (**a**) The stringent scoring MR plot shows the analysis as performed previously[1], taking the least significant M score and lowest R score from the comparisons. Using cut-offs of M > 4, R > 0.81 this high stringency analysis had an estimated false discovery rate (FDR) of <1% and yielded three hits – ATG9A, SERINC1 and SERINC3 (marked in yellow) – which we subsequently validated as bona fide AP-4 cargo proteins[1]. (**b**) In this study, we reanalysed the data with a high sensitivity scoring method, this time taking the median M score and the mean R score for each protein. With the same movement cut-off of M > 4 and a stringent reproducibility cut-off of R > 0.92, there were 11 hits with an estimated overall FDR of approximately 20%. This includes the three known AP-4 cargo proteins (yellow) and 8 new hits marked in red (see Fig. 1a for details). For the 8 new hits, the FDR is thus estimated at 25%. The sensitive scoring in **b** results in a larger spread of M scores and allows the identification of hits that did not meet the movement cut-off in **a**, but still undergo highly reproducible shifts in the KO cells. However, the higher FDR indicates that some false positives are expected. (**c & d**) Unlike DAGLB (Fig. 1d), the shift profiles (abundance distribution profiles from knockout maps subtracted from the profiles from control maps) of (**c**) PTPN9 and (**d**) LNPEP do not correlate with the shift profiles of the known AP-4 vesicle cargos, ATG9A, SERINC1 and SERINC3. Thus, they are unlikely to be AP-4 vesicle proteins.

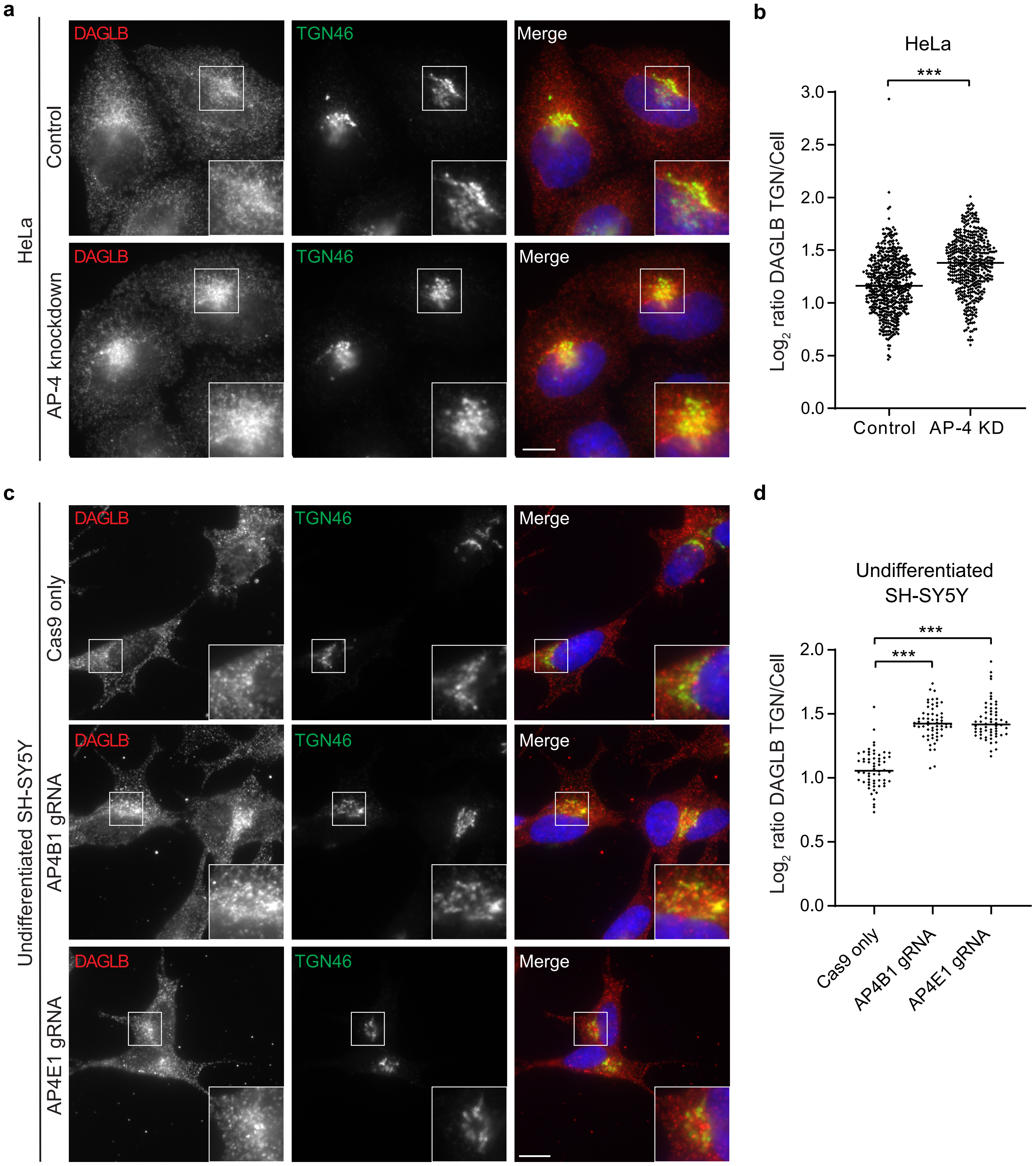
a
HeLa
Control
AP-4 knockdown
DAGLB
TGN46
Merge
b
HeLa
***
Log2 ratio DAGLB TGN/Cell
3.0
2.5
2.0
1.5
1.0
0.5
0.0
Control
AP-4 KD
c
Undifferentiated SH-SY5Y
Cas9 only
AP4B1 gRNA
AP4E1 gRNA
d
Undifferentiated SH-SY5Y
***
***
2.0
1.5
1.0
0.5
0.0
Cas9 only
AP4B1 gRNA
AP4E1 gRNA

Supplementary Figure 2. DAGLB accumulates at the trans-Golgi network (TGN) in AP-4 knockdown (KD) HeLa and undifferentiated AP-4-depleted SH-SY5Y cells. (**a**) Widefield imaging of immunofluorescence double labelling of DAGLB (red) and TGN46 (green) in HeLa cells transfected with a non-targeting siRNA (control) or with siRNA to knock down AP-4. In the merged image, DAPI labelling of the nucleus is also shown (blue). Scale bar: 10 µm. (**b**) Quantification of the ratio of DAGLB labelling intensity between the TGN and the rest of the cell, in the cells shown in **a**. The experiment was performed in biological triplicate and the graph shows combined replicate data: each datapoint indicates the $\log_2$ ratio for an individual cell (horizontal bar indicates median; n = 550 cells for control; n = 466 cells for KD; examined over 3 independent experiments). Data were subjected to a two-tailed Mann–Whitney U-test: ***$p \leq 0.001$ ($p = 9.5 \times 10^{-30}$). (**c**) Widefield imaging of immunofluorescence double labelling of DAGLB (red) and TGN46 (green) in control (parental Cas9-expressing), AP4B1-depleted and AP4E1-depleted undifferentiated SH-SY5Y cells. In the merged image, DAPI labelling of the nucleus is also shown (blue). Scale bar: 10 µm. (**d**) Quantification of the ratio of DAGLB labelling intensity between the TGN and the rest of the cell, in the cells shown in **c**. The experiment was performed in biological triplicate and the graph shows combined replicate data: each datapoint indicates the $\log_2$ ratio calculated from a single image (horizontal bar indicates median; n = 60 images per cell line; examined over 3 independent experiments). Data were subjected to a Kruskal-Wallis test with Dunn's Multiple Comparison Test for significance: ***$p \leq 0.001$ (Cas9 vs AP4B1: $p = 2.2 \times 10^{-18}$; Cas9 vs AP4E1: $p = 1.5 \times 10^{-18}$). Source data are provided as a Source Data file.

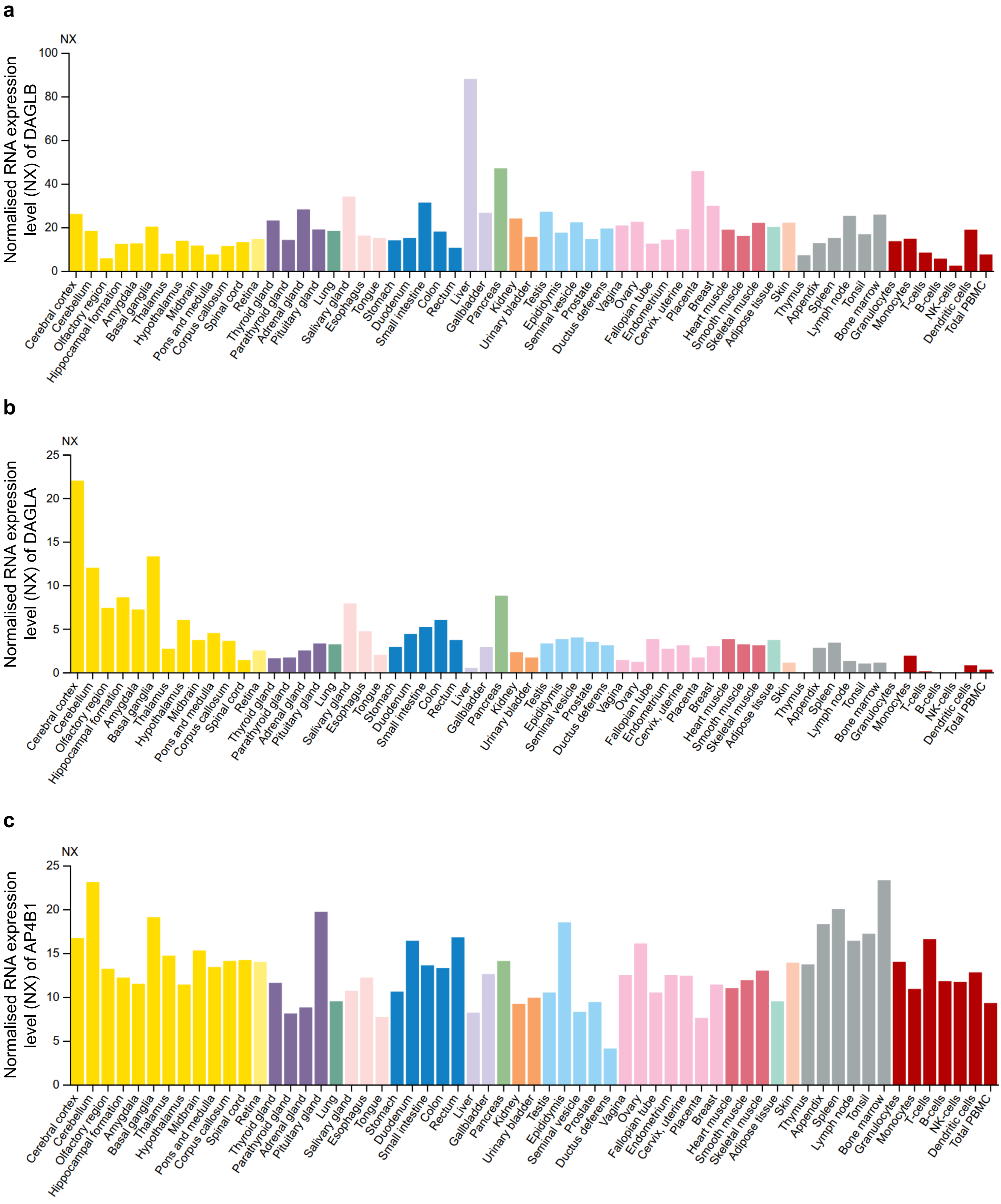

Supplementary Figure 3. DAGLB is expressed with low tissue specificity, like AP-4, while DAGLA expression is most pronounced in the brain. Consensus normalised RNA expression (NX) levels of (**a**) DAGLB, (**b**) DAGLA and (**c**) AP4B1 for 55 tissue types and 6 blood cell types, from the Human Protein Atlas[2]. Tissues are grouped and coloured according to shared functional features. Image credit: Human Protein Atlas [https://www.proteinatlas.org/ENSG00000164535-DAGLB/tissue; https://www.proteinatlas.org/ENSG00000134780-DAGLA/tissue; https://www.proteinatlas.org/ENSG00000134262-AP4B1/tissue].

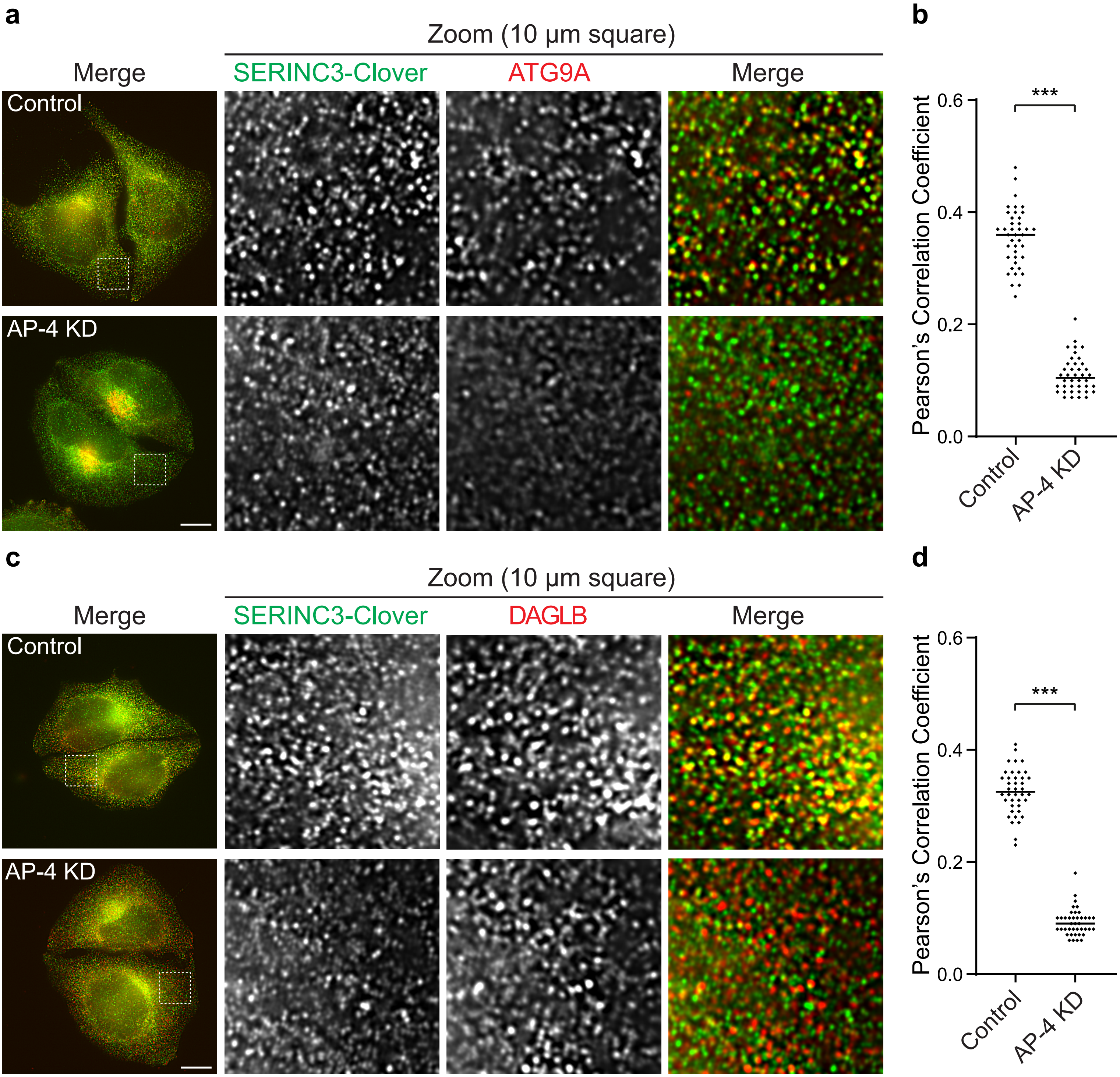

Supplementary Figure 4. DAGLB and ATG9A colocalise with SERINC3 in an AP-4-dependent manner. HeLa cells tagged endogenously with Clover (modified GFP) at the C-terminus of SERINC3 were transfected with siRNA to knock down (KD) AP-4, or were transfected with a non-targeting siRNA (Control). (**a**) Super resolution structured illumination microscopy (SR-SIM) was used to image SERINC3-Clover (via anti-GFP; green) and anti-ATG9A (red). Representative images show the whole field of view and a zoomed image of a peripheral $10 \times 10\ \mu m^2$ square. ATG9A and SERINC3 colocalised in small puncta throughout the cytoplasm in control cells, but not in AP-4-depleted cells. Scale bar: 10 μm. (**b**) Quantification of colocalisation between SERINC3-Clover and ATG9A in control and AP-4 knockdown (KD) cells, using Pearson's Correlation Coefficient (PCC). The experiment was performed in biological duplicate and the graph shows combined replicate data: each datapoint indicates the PCC for an individual cell (horizontal bar indicates median; n = 40 cells per condition, examined across 2 independent experiments). Data were subjected to a two-tailed Mann–Whitney U-test: ***$p \leq 0.001$ ($p = 1.9 \times 10^{-23}$). (**c**) SR-SIM was used to image SERINC3-Clover (via anti-GFP; green) and anti-DAGLB (red), as in **a**. Like ATG9A, DAGLB colocalised with SERINC3 in small puncta throughout the cytoplasm in control cells, but not in AP-4-depleted cells. (**d**) Quantification of colocalisation between SERINC3-Clover and DAGLB in control and AP-4 KD cells, using PCC. The experiment was performed in biological duplicate and the graph shows combined replicate data: each datapoint indicates the PCC for an individual cell (horizontal bar indicates median; n = 40 cells per condition, examined across 2 independent experiments). Data were subjected to a two-tailed Mann–Whitney U-test: ***$p \leq 0.001$ ($p = 1.9 \times 10^{-23}$). Source data are provided as a Source Data file.

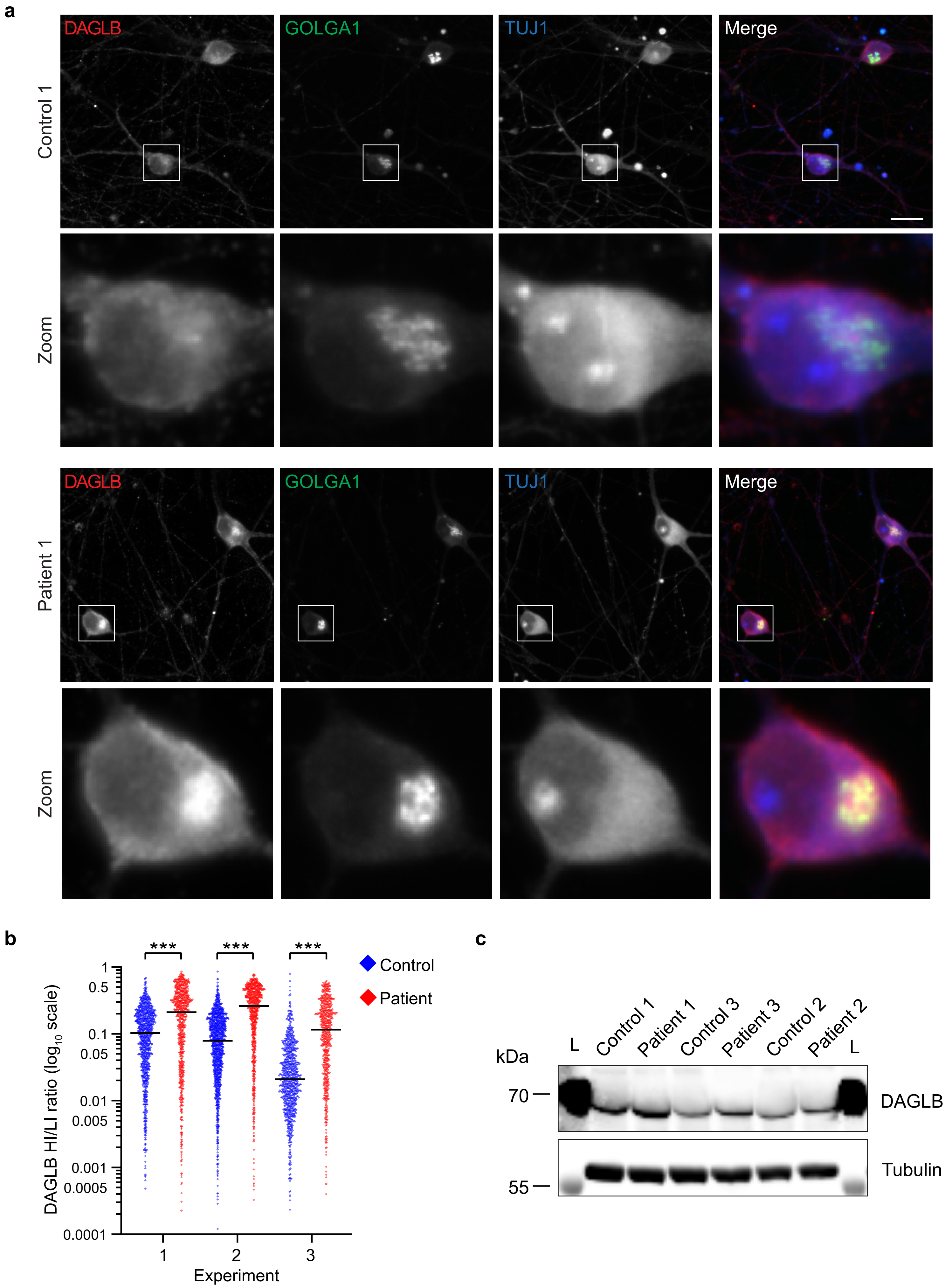
a
DAGLB
GOLGA1
TUJ1
Merge
Control 1
Zoom
Patient 1
Zoom
b
***
***
***
Control
Patient
DAGLB HI/LI ratio (log_{10} scale)
1
0.5
0.1
0.05
0.01
0.005
0.001
0.0005
0.0001
1
2
3
Experiment
c
kDa
L
Control 1
Patient 1
Control 3
Patient 3
Control 2
Patient 2
L
70
55
DAGLB
Tubulin

Supplementary Figure 5. Assessment of DAGLB localisation and abundance in iPSC-derived neurons from patients with AP-4 deficiency syndrome. Induced pluripotent stem cells (iPSCs) from patients with AP4B1-associated AP-4 deficiency syndrome (SPG47) and their unaffected same sex heterozygous parents (control) were differentiated into cortical neurons. (**a**) Representative images from the high-throughput imaging assay shown in Fig. 6c. iPSC-derived neurons in 96-well plates were labelled with antibodies against DAGLB (red), GOLGA1 (a TGN marker; green) and TUJ1 (a marker to distinguish neurons from co-cultured astrocytes; blue), and imaged on an automated confocal microscope. The zoomed regions show an increase in DAGLB signal in the juxtanuclear area (high intensity area) of the patient neurons (patient 1), which overlaps with the TGN marker GOLGA1. Scale bar: 20 µm. (**b**) High-throughput confocal imaging was used to assay the distribution of DAGLB in iPSC-derived neurons from patient 2 and their matched control. Neurons in 96-well plates were labelled with antibodies against DAGLB, GOLGA1 (a TGN marker) and TUJ1. The ratio between the area of high intensity (HI; overlaps with TGN) and low intensity (LI) DAGLB labelling was quantified from three differentiations per cell line (biological triplicate; plotted separately): each datapoint indicates the ratio for an individual cell, plotted on a $\log_{10}$ scale (horizontal bar indicates median; experiment 1/2/3: n = 983/1302/855 cells for control; n = 780/795/658 cells for patient). Data were subjected to a two-tailed Mann-Whitney U-test for comparison of the patient and control within each differentiation: ***$p \leq 0.001$ (1: $p = 5.7 \times 10^{-26}$; 2: $p = 6.4 \times 10^{-83}$; 3: $p = 1.0 \times 10^{-79}$). Comparable results for another SPG47 patient (patient 1) are shown in Fig. 6c. (**c**) Western blot for DAGLB using whole cell lysates from iPSC-derived neurons from three patients with SPG47 and their matched controls. Alpha-tubulin serves as a loading control. L: molecular weight markers. Representative of four independent experiments (quantified in Fig. 6d). Source data are provided as a Source Data file.

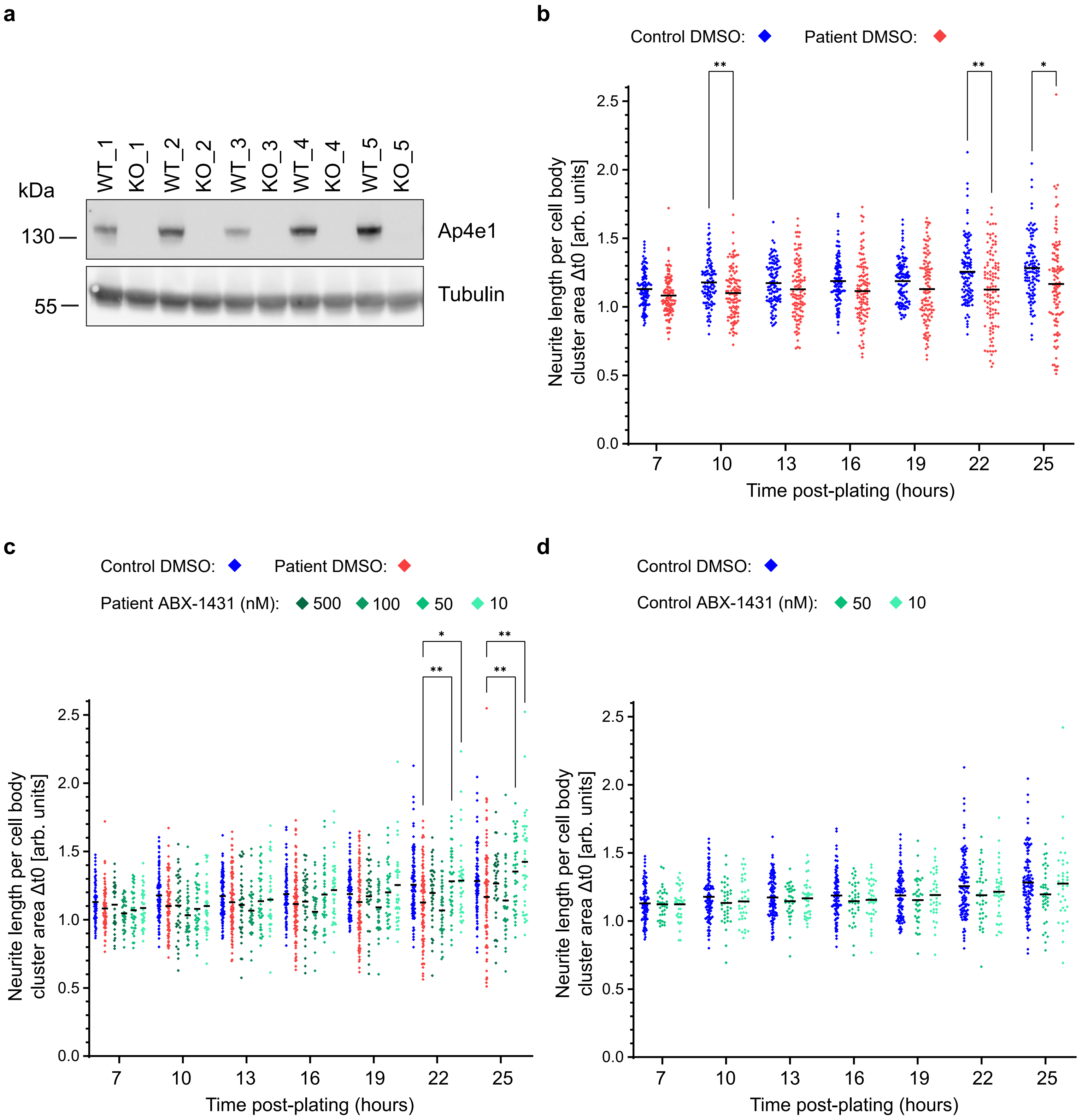

a
kDa
WT_1
KO_1
WT_2
KO_2
WT_3
KO_3
WT_4
KO_4
WT_5
KO_5
130
55
Ap4e1
Tubulin
b
Control DMSO:
Patient DMSO:
Neurite length per cell body cluster area Δt0 [arb. units]
Time post-plating (hours)
c
Patient ABX-1431 (nM): 500 100 50 10
d
Control ABX-1431 (nM): 50 10

Supplementary Figure 6. 2-AG and AA are reduced in AP-4 knockout brains and MGLL inhibition rescues impaired neurite outgrowth in AP-4-deficient neurons. (**a**) Western blot for AP4E1 from the brains of mice used for the lipid analyses in Fig. 7b-d, to confirm the AP-4 knockout genotype. Alpha-tubulin serves as a loading control. WT: wild-type; KO: *Ap4e1* knockout. (**b-d**) Neurite outgrowth was assayed in iPSC-derived cortical neurons from a patient with AP4B1-associated AP-4 deficiency syndrome (SPG47; patient 2) and their unaffected same sex heterozygous parent (control), using automated live cell imaging. Neurons were cultured in the presence of DMSO (vehicle control) or the MGLL inhibitor ABX-1431 at 10, 50, 100 or 500 nM (the highest two doses were administered only to the patient neurons). Neurons were monitored from 4 h post-plating, with images captured every 3 h until 25 h post-plating. Graphs show neurite length per image over time, normalised to cell body cluster area, for two separate differentiations per cell line. Data are shown relative to normalised neurite length at *t*0. The same data are shown for the control plus DMSO condition in **b-d** and for the patient plus DMSO condition in **b** and **d**. Similar results were observed for another patient iPSC neuron line (see Fig. 7f-h). (**b**) Average neurite length of patient neurons was reduced compared to control neurons at all time points. Per group, $n = 108$ images from two biological replicates were analysed. Data were subjected to a two-way repeated measures ANOVA with Šídák's multiple comparisons test for comparisons at each time point between control and patient: $^{**}p \leq 0.01$; $^{*}p \leq 0.05$. (**c**) Average neurite length of patient neurons was rescued by treatment with 10 or 50 nM ABX-1431, whereas neurite length was not improved by the higher doses. Per treatment group, $n = 36$ images from two biological replicates were analysed. Data were subjected to a two-way repeated measures ANOVA with Dunnett's multiple comparisons test for comparisons at each time point between each dose of ABX-1431 and the patient plus DMSO control: $^{**}p \leq 0.01$; $^{*}p \leq 0.05$. The control plus DMSO condition is shown for reference, but was not included in the statistical analysis. (**d**) Average neurite length of control neurons was not affected by treatment with 10 or 50 nM ABX-1431. Per treatment group, n = 36 images from two biological replicates were analysed. Data were subjected to a two-way repeated measures ANOVA with Dunnett's multiple comparisons test for comparisons at each time point between each dose of ABX-1431 and the DMSO control. Source data are provided as a Source Data file.

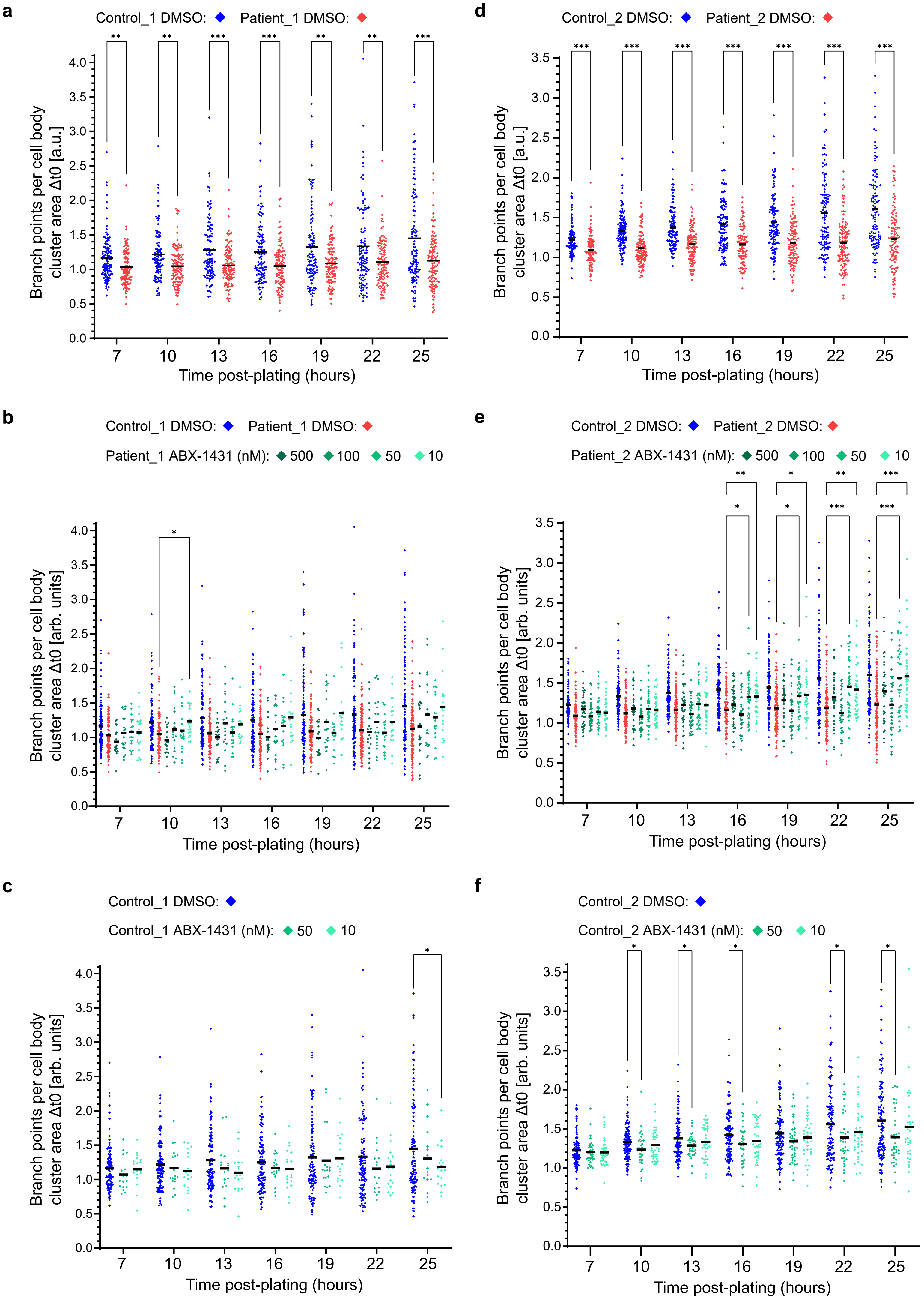
a
Control_1 DMSO:
Patient_1 DMSO:
** ** *** *** ** ** ***
Branch points per cell body cluster area Δt0 [a.u.]
4.0
3.5
3.0
2.5
2.0
1.5
1.0
0.5
0.0
7 10 13 16 19 22 25
Time post-plating (hours)
d
Control_2 DMSO:
Patient_2 DMSO:
*** *** *** *** *** *** ***
Branch points per cell body cluster area Δt0 [a.u.]
3.5
3.0
2.5
2.0
1.5
1.0
0.5
0.0
7 10 13 16 19 22 25
Time post-plating (hours)
b
Control_1 DMSO:
Patient_1 DMSO:
Patient_1 ABX-1431 (nM): 500 100 50 10
*
Branch points per cell body cluster area Δt0 [arb. units]
4.0
3.5
3.0
2.5
2.0
1.5
1.0
0.5
0.0
7 10 13 16 19 22 25
Time post-plating (hours)
e
Control_2 DMSO:
Patient_2 DMSO:
Patient_2 ABX-1431 (nM): 500 100 50 10
** * ** ***
* * *** ***
Branch points per cell body cluster area Δt0 [arb. units]
3.5
3.0
2.5
2.0
1.5
1.0
0.5
0.0
7 10 13 16 19 22 25
Time post-plating (hours)
c
Control_1 DMSO:
Control_1 ABX-1431 (nM): 50 10
*
Branch points per cell body cluster area Δt0 [arb. units]
4.0
3.5
3.0
2.5
2.0
1.5
1.0
0.5
0.0
7 10 13 16 19 22 25
Time post-plating (hours)
f
Control_2 DMSO:
Control_2 ABX-1431 (nM): 50 10
* * * * *
Branch points per cell body cluster area Δt0 [arb. units]
3.5
3.0
2.5
2.0
1.5
1.0
0.5
0.0
7 10 13 16 19 22 25
Time post-plating (hours)

Supplementary Figure 7. MGLL inhibition rescues the neurite branching defect in AP-4-deficient neurons. Neurite outgrowth was assayed in iPSC-derived cortical neurons from two patients with AP4B1-associated AP-4 deficiency syndrome (SPG47) and their unaffected same sex heterozygous parents (control), using automated live cell imaging. Neurons were cultured in the presence of DMSO (vehicle control) or the MGLL inhibitor ABX-1431 at 10, 50, 100 or 500 nM (the highest two doses were administered only to the patient neurons). Neurons were monitored from 4 h post-plating, with images captured every 3 h until 25 h post-plating. Graphs show number of neurite branch points per image over time, normalised to cell body cluster area, for two separate differentiations per cell line. Data are shown relative to normalised branch point number at $t0$. Panels **a**, **b** and **c** show data for patient 1 and their matched control and panels **d**, **e** and **f** show data for patient 2 and their matched control. (**a** & **d**) Average number of neurite branch points of patient neurons was reduced compared to control neurons at all time points. Per group, n = 108 images from two biological replicates were analysed. Data were subjected to a two-way repeated measures ANOVA with Šídák's multiple comparisons test for comparisons at each time point between control and patient: ***$p \leq 0.001$; **$p \leq 0.01$. (**b** & **e**) Average number of neurite branch points of patient neurons was rescued by treatment with 10 or 50 nM ABX-1431, but was not improved by the higher doses. The increase in number of branch points was significant in the analysis of patient 2 (shown in **e**), and the data for patient 1 (shown in **b**) followed the same trend, despite not reaching statistical significance. Per treatment group, n = 18 images (patient 1) and n = 36 images (patient 2) from two biological replicates were analysed. Data were subjected to a two-way repeated measures ANOVA with Dunnett's multiple comparisons test for comparisons at each time point between each dose of ABX-1431 and the patient plus DMSO control: ***$p \leq 0.001$; **$p \leq 0.01$; *$p \leq 0.05$. The control plus DMSO condition is shown for reference but was not included in the statistical analysis. (**c** & **f**) Average number of neurite branch points of control neurons was not increased by treatment with 10 or 50 nM ABX-1431. However, at 50 nM there was a reduction in the number of branch points at several time points, suggesting that 2-AG levels must be finely balanced to ensure correct neurite outgrowth. Per treatment group, n = 18 images (patient 1) and n = 36 images (patient 2) from two biological replicates were analysed. Data were subjected to a two-way repeated measures ANOVA with Dunnett's multiple comparisons test for comparisons at each time point between each dose of ABX-1431 and the DMSO control: *$p \leq 0.05$. Source data are provided as a Source Data file.

Fig. S5c: DAGLB & Tubulin

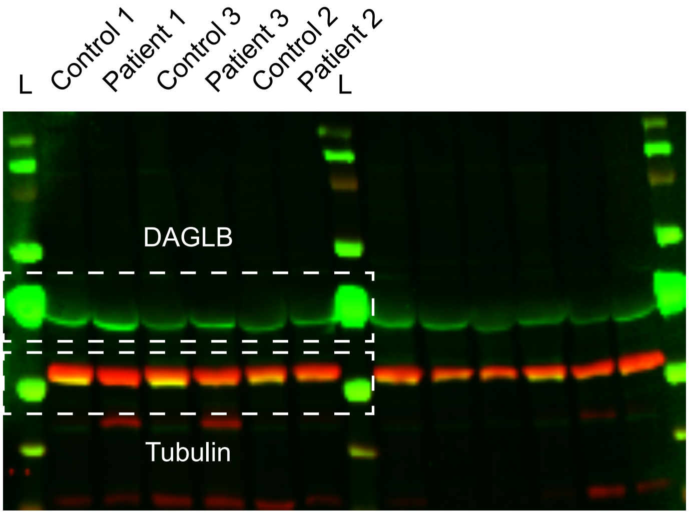

Fig. S6a: Ap4e1

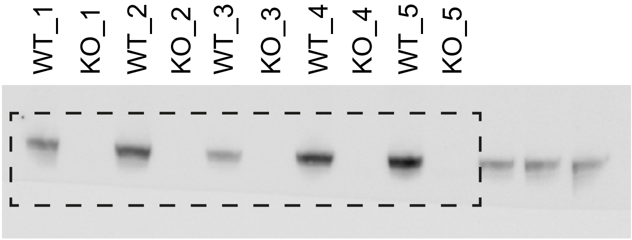

Fig. S6a: Tubulin

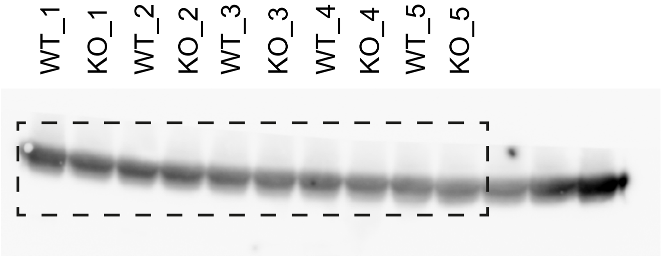

Supplementary Figure 8. Uncropped images of Western blots shown in Supplementary Fig. 5c and 6a. The relevant lanes, antibodies and figures in which they appear are indicated on each blot.

Supplementary Table 1. Summary of AP-4 vesicle cargo and accessory proteins supported by unbiased proteomic approaches.

Gene name	Role	Proteomic evidence	Other evidence	References
**ATG9A**	Cargo	Maps, IP, BioID, vesicle fraction	Microscopy, *in vitro* interaction	Davies et al. 2018; Ivankovic et al. 2020; Mattera et al. 2015/2017[1,3–5]
**SERINC1**	Cargo	Maps, IP, vesicle fraction	Microscopy	Davies et al. 2018[1]
**SERINC3**	Cargo	Maps, IP, vesicle fraction	Microscopy	Davies et al. 2018[1]
**DAGLB**	Cargo	Maps, IP, vesicle fraction	Microscopy	This study; Davies et al. 2018[1]
**TEPSIN**	Accessory (unknown function)	IP, BioID, vesicle fraction	Microscopy, *in vitro* interaction	Borner et al. 2012; Davies et al. 2018; Frazier et al. 2016; Mattera et al. 2015[1,5–7]
**RUSC1**	Accessory (anterograde transport adaptor)	Vesicle fraction, membrane fraction		Davies et al. 2018[1]
**RUSC2**	Accessory (anterograde transport adaptor)	BioID, vesicle fraction	Microscopy, *in vitro* interaction	Davies et al. 2018[1]
**HOOK1**	Accessory (retrograde transport adaptor, FHF complex)	IP, BioID	*In vitro* interaction	Davies et al. 2018; Mattera et al. 2020[1,8]
**HOOK2**	Accessory (retrograde transport adaptor, FHF complex)	None, but inferred from HOOK1/3	Microscopy, *in vitro* interaction	Mattera et al. 2020[8]
**HOOK3**	Accessory (retrograde transport adaptor, FHF complex)	IP, BioID	*In vitro* interaction	Davies et al. 2018; Mattera et al. 2020[1,8]
**FAM160A1**	Accessory (retrograde transport adaptor, FHF complex)	IP, BioID	Microscopy	Davies et al. 2018; Mattera et al. 2020[1,8]
**FAM160A2**	Accessory (retrograde transport adaptor, FHF complex)	IP, BioID	Microscopy, *in vitro* interaction	Davies et al. 2018; Mattera et al. 2015/2020[1,5,8]
**AKTIP**	Accessory (retrograde transport adaptor, FHF complex)	IP, BioID		Davies et al. 2018; Mattera et al. 2020[1,8]
**AAGAB**	Accessory (unknown function, also binds AP-1 and AP-2)	IP, BioID		Davies et al. 2018[1]

Inclusion criteria: evidence from at least two orthogonal experiments, where at least one was an unbiased proteomic approach assaying AP-4-association with endogenous proteins.

Maps: protein had a significantly altered distribution in AP-4 knockout cells in 'Dynamic Organellar Maps' fractionation-based spatial proteomics; IP: protein co-immunoprecipitated with the AP-4 complex; BioID: protein was specifically biotinylated by AP-4 complex baits; vesicle fraction: protein had an altered abundance in vesicle fractions prepared from AP-4-deficient cells; membrane fraction: protein had an altered abundance in membrane fractions prepared from AP-4 knockout cells. Integral membrane proteins of AP-4 vesicles are considered cargo; accessory factors are cytosolic proteins that transiently associate with AP-4 vesicles. The tetrameric AP-4 complex itself (AP4E1, AP4B1, AP4M1, AP4S1) is not listed here.

Supplementary references

1. Davies, A. K. *et al.* AP-4 vesicles contribute to spatial control of autophagy via RUSC-dependent peripheral delivery of ATG9A. *Nat. Commun.* **9**, 3958 (2018).

2. Uhlén, M. *et al.* Tissue-based map of the human proteome. *Science* **347** (6220), 1260419 (2015).

3. Ivankovic, D. *et al.* Axonal autophagosome maturation defect through failure of ATG9A sorting underpins pathology in AP-4 deficiency syndrome. *Autophagy* **16**, 391–407 (2020).

4. Mattera, R., Park, S. Y., De Pace, R., Guardia, C. M. & Bonifacino, J. S. AP-4 mediates export of ATG9A from the trans-Golgi network to promote autophagosome formation. *Proc. Natl. Acad. Sci.* **114**, E10697–E10706 (2017).

5. Mattera, R., Guardia, C. M., Sidhu, S. S. & Bonifacino, J. S. Bivalent motif-ear interactions mediate the association of the accessory protein tepsin with the AP-4 adaptor complex. *J. Biol. Chem.* **290**, 30736–30749 (2015).

6. Borner, G. H. H. *et al.* Multivariate proteomic profiling identifies novel accessory proteins of coated vesicles. *J. Cell Biol.* **197**, 141–160 (2012).

7. Frazier, M. N. *et al.* Molecular Basis for the Interaction Between AP4 beta4 and its Accessory Protein, Tepsin. *Traffic* **17**, 400–415 (2016).

8. Mattera, R., Williamson, C. D., Ren, X. & Bonifacinoa, J. S. The FTS-Hook-FHIP (FHF) complex interacts with AP-4 to mediate perinuclear distribution of AP-4 and its cargo ATG9A. *Mol. Biol. Cell* **31**, 963–979 (2020).
