# Supplementary material for: AP-4-mediated axonal transport controls endocannabinoid production in neurons
Source: Nat Commun. 2022 Feb 25;13:1058. doi: 10.1038/s41467-022-28609-w (PMC8881493; doi:10.1038/s41467-022-28609-w)
Supplement: Supplementary file 3 — Reporting Summary [file 41467_2022_28609_MOESM3_ESM.pdf]

nature research

Corresponding author(s): Georg H H Borner, Alexandra K Davies

Last updated by author(s): Nov 18, 2021

Reporting Summary

Nature Research wishes to improve the reproducibility of the work that we publish. This form provides structure for consistency and transparency in reporting. For further information on Nature Research policies, see our Editorial Policies and the Editorial Policy Checklist.

Statistics

For all statistical analyses, confirm that the following items are present in the figure legend, table legend, main text, or Methods section.

n/a	Confirmed	
☐	☒	The exact sample size (*n*) for each experimental group/condition, given as a discrete number and unit of measurement
☐	☒	A statement on whether measurements were taken from distinct samples or whether the same sample was measured repeatedly
☐	☒	The statistical test(s) used AND whether they are one- or two-sided<br>*Only common tests should be described solely by name; describe more complex techniques in the Methods section.*
☐	☒	A description of all covariates tested
☐	☒	A description of any assumptions or corrections, such as tests of normality and adjustment for multiple comparisons
☐	☒	A full description of the statistical parameters including central tendency (e.g. means) or other basic estimates (e.g. regression coefficient) AND variation (e.g. standard deviation) or associated estimates of uncertainty (e.g. confidence intervals)
☐	☒	For null hypothesis testing, the test statistic (e.g. *F*, *t*, *r*) with confidence intervals, effect sizes, degrees of freedom and *P* value noted<br>*Give P values as exact values whenever suitable.*
☒	☐	For Bayesian analysis, information on the choice of priors and Markov chain Monte Carlo settings
☐	☒	For hierarchical and complex designs, identification of the appropriate level for tests and full reporting of outcomes
☐	☒	Estimates of effect sizes (e.g. Cohen's *d*, Pearson's *r*), indicating how they were calculated

Our web collection on statistics for biologists contains articles on many of the points above.

Software and code

Policy information about availability of computer code

Data collection	Raw microscopy images were acquired using commercially available software - either LAS X (Leica Application Software X) version 3.5.5.19976, Zeiss ZEN 2, MetaXpress version 6.7.0.211 or IncuCyte Controller Version 2020B, as detailed in the Methods section.<br>Raw mass spectrometry data for lipid analysis were acquired with Bruker Otofcontrol version 6.
Data analysis	Colocalisation analysis was performed in ImageJ version 2.1.0 (Schneider et al., 2012) using the Coloc2 plugin (FIJI package distribution) and a custom script, available at https://zenodo.org/ (pending upload). Analysis of DAGLB distribution was performed in the software CellProfiler (Mcquin et al., 2018), version 3.1.9, and pipeline files for each cell type are available at https://zenodo.org/ (pending upload). Analysis of high-throughput confocal imaging of DAGLB distribution was performed using a customised image analysis pipeline in MetaXpress version 6.7.0.211 (Molecular Devices). Neurite outgrowth was quantified using the Incucyte® Neurotrack Analysis Software Module in IncuCyte Controller Version 2020B (Sartorius, Cat# 9600-0010).<br>Mass spectrometry proteomics data were analysed using Perseus version 1.6 (Tyanova et al., 2016). Processing and analysis of the raw files for lipid analysis were performed with MetaboScape 2021 version (Bruker Daltonics, Germany).<br>Statistical analyses were performed in GraphPad Prism version 9.1.2 for Windows.

For manuscripts utilizing custom algorithms or software that are central to the research but not yet described in published literature, software must be made available to editors and reviewers. We strongly encourage code deposition in a community repository (e.g. GitHub). See the Nature Research guidelines for submitting code & software for further information.

Data

Policy information about availability of data

All manuscripts must include a data availability statement. This statement should provide the following information, where applicable:

- Accession codes, unique identifiers, or web links for publicly available datasets
- A list of figures that have associated raw data
- A description of any restrictions on data availability

The raw mass spectrometry proteomics data associated with Fig. 1 and Supplementary Fig. 1 were previously published (Davies et al., 2018) and are available from the ProteomeXchange Consortium via the PRIDE partner repository with the dataset identifier PXD010103 [http://proteomecentral.proteomexchange.org/cgi/GetDataset?ID=PXD010103]. The raw mass spectrometry lipidomics data generated in this study (Fig. 7b-d) have been deposited in the MASS Spectrometry Interactive Virtual Environment (MassIVE) and are accessible via the identifier MSV000088020 [ftp://massive.ucsd.edu/MSV000088020/]. The imaging data generated in this study have been deposited on Zenodo and are accessible via the identifiers 5696988 [https://zenodo.org/record/5696988], 5698395 [https://doi.org/10.5281/zenodo.5698395] and 5644233 [https://doi.org/10.5281/zenodo.5644233]. The RNA expression data shown in Supplementary Fig. 3 is publicly available from the Human Protein Atlas via the following links: DAGLB [https://www.proteinatlas.org/ENSG00000164535-DAGLB/tissue], DAGLA [https://www.proteinatlas.org/ENSG00000134780-DAGLA/tissue], AP4B1 [https://www.proteinatlas.org/ENSG00000134262-AP4B1/tissue]. Source data are provided with this paper.

Field-specific reporting

Please select the one below that is the best fit for your research. If you are not sure, read the appropriate sections before making your selection.

- [x] Life sciences
- [ ] Behavioural & social sciences
- [ ] Ecological, evolutionary & environmental sciences

For a reference copy of the document with all sections, see nature.com/documents/nr-reporting-summary-flat.pdf

Life sciences study design

All studies must disclose on these points even when the disclosure is negative.

Sample size	No statistical method was used to predetermine sample size. For the SR-SIM colocalisation analyses shown in Fig. 5b, d and S4b, d, sample sizes were chosen based on our previously published colocalisation analyses of SERINCs and ATG9A (Davies et al., 2018). For the high-throughput analyses of DAGLB localisation (Fig. 6c and S5b) and the automated live cell analyses of neurite outgrowth (Fig. 7f-h, S6b-d and S7), sample sizes were chosen based on our previously published high-throughput analyses of ATG9A localisation and analyses of neurite outgrowth in AP-4-deficient iPSC neurons (Behne et al., 2020).
Data exclusions	Generally, quantitative image data underwent threshold filtering prior to analysis, as described in detail in the methods, and edge cells were excluded, with the aim to avoid unreliable or partial measurements near the detection limit of the method. Importantly, no data were ever excluded from the analyses as outliers.
Replication	Fig. 1a: Dynamic Organellar Maps of AP4B1 knockout (KO) and AP4E1 KO HeLa cells were compared to maps of wild-type HeLa cells (each in duplicate, totalling 4 comparisons).<br>Fig. 1e: High-sensitivity low-detergent immunoprecipitations (IP) from HeLa cells stably expressing the AP-4 associated protein TEPSIN-GFP, n = 3.<br>Fig. 2b: Quantification of the ratio of DAGLB labelling intensity between the TGN and the rest of the cell, in wild-type (WT), AP4B1 KO, and AP4B1 KO HeLa cells stably expressing AP4B1, n = 655 cells for WT; n = 588 cells for KO; n = 586 cells for Rescue; examined over 3 independent experiments.<br>Fig. 2d: Quantification of the ratio of DAGLB labelling intensity between the TGN and the rest of the cell, in control (parental Cas9-expressing), AP4B1-depleted and AP4E1-depleted neuronally-differentiated SH-SY5Y cells, n = 50 images for Cas9 only; n = 47 images for AP4B1 gRNA; n = 50 images for AP4E1 gRNA; examined over 3 independent experiments.<br>Fig. 2f: Quantification of DAGLB from Western blots, n = 4 blots per cell line, and three separate differentiations.<br>Fig. 3: Widefield imaging of HeLa cells stably expressing RUSC2-GFP (green), labelled with anti-DAGLB (red). Top panel, wild-type; middle panel, AP4B1 knockout; lower panel, AP4B1 knockout with transient expression of AP4B1 (rescue), images are representative of at least 20 images per condition, including three independent rescue transfections.<br>Fig. 4a, b: Widefield imaging of wild-type HeLa or HeLa cells stably expressing GFP-RUSC2 (blue), transiently expressing HA-DAGLB or HA-DAGLA, images are representative of at least 18 images per condition, including three independent transfections with microscopy performed independently for each.<br>Fig. 5b, d, S4b, d: Quantification of colocalisation between SERINC1-Clover/SERINC3-Clover and ATG9A/DAGLB in control and AP-4 knockdown (KD) cells, n = 40 cells per condition, examined across 2 independent experiments.<br>Fig. 6b: Quantification of the ratio of DAGLB labelling intensity between the TGN and the rest of the cell, in iPSC neurons from patient 1 and their matched control, the experiment was performed in technical triplicate, n = 227 cells for Control; n = 129 cells for Patient.<br>Fig. 6c: High-throughput confocal imaging was used to assay the distribution of DAGLB in iPSC-derived neurons from patient 1 and their matched control, quantified from three differentiations per cell line (biological triplicate), experiment 1/2/3: n = 417/786/999 cells for control; n = 306/464/339 cells for patient.<br>Fig. 6d: Western blotting was used to quantify the level of DAGLB in whole cell lysates from iPSC-derived neurons from three patients with SPG47 and their unaffected same sex heterozygous parents. Data are from n = 4 differentiations per cell line.<br>Fig. 6f: High-throughput confocal imaging was used to assay the density of DAGLB puncta in axons of iPSC neurons from patient 1 and their matched control, n = 324 images in the control group and n = 405 images in the patient group, covering 21902 and 25253 axon segments respectively.

Fig. 7b-d: Mass spectrometry-based quantification of (b) 2-AG, (c) AA and (d) 1-stearoyl-2-arachidonoyl-sn-glycerol (SAG), from wild-type (WT) and Ap4e1 knockout (KO) mouse brains, n = 5 animals per group.
Fig. 7f, S6b, S7a, d: Neurite growth of patient neurons compared to control neurons, per group, n = 108 images from two biological replicates were analysed.
Fig. 7g, h, S7b, c: Neurite growth of patient 1/ control 1 neurons with treatment with ABX-1431, n = 18 images from two biological replicates were analysed per group.
Fig. S2b: Quantification of the ratio of DAGLB labelling intensity between the TGN and the rest of the cell, in HeLa cells transfected with a non-targeting siRNA (control) or with siRNA to knock down AP-4, n = 550 cells for control; n = 466 cells for KD; examined over 3 independent experiments.
Fig. S2d: Quantification of the ratio of DAGLB labelling intensity between the TGN and the rest of the cell, in control (parental Cas9-expressing), AP4B1-depleted and AP4E1-depleted undifferentiated SH-SY5Y cells, n = 60 images per cell line; examined over 3 independent experiments.
Fig. S5b: High-throughput confocal imaging was used to assay the distribution of DAGLB in iPSC-derived neurons from patient 2 and their matched control, quantified from three differentiations per cell line (biological triplicate), experiment 1/2/3: n = 983/1302/855 cells for control; n = 780/795/658 cells for patient.
Fig. S6c, d, S7e, f: Neurite growth of patient 2/control 2 neurons with treatment with ABX-1431, n = 36 images from two biological replicates were analysed per group.

Randomization

This is not relevant to the study, as we were only comparing genetically different cells/animals. No treatment of subjects was performed.

Blinding

The investigators were not blinded to allocation during experiments and outcome assessment. However, all image analyses were automated to avoid investigator bias. Additionally, objective imaging was achieved by focusing the microscope on an invariant marker protein first, without seeing the fluorescence channel of the protein of experimental relevance. Images were then collected for both channels in the same field of view. Thus, although the experimenter was not blinded to sample ID, he or she was effectively blinded to the critical data during collection. This is described in the methods.

Reporting for specific materials, systems and methods

We require information from authors about some types of materials, experimental systems and methods used in many studies. Here, indicate whether each material, system or method listed is relevant to your study. If you are not sure if a list item applies to your research, read the appropriate section before selecting a response.

Materials & experimental systems

n/a	Involved in the study
☐	☒ Antibodies
☐	☒ Eukaryotic cell lines
☒	☐ Palaeontology and archaeology
☐	☒ Animals and other organisms
☒	☐ Human research participants
☒	☐ Clinical data
☒	☐ Dual use research of concern

Methods

n/a	Involved in the study
☒	☐ ChIP-seq
☒	☐ Flow cytometry
☒	☐ MRI-based neuroimaging

Antibodies

Antibodies used

The following antibodies were used in this study: mouse anti-alpha tubulin 1:1000 for WB (clone DM1A, Sigma-Aldrich Cat# T9026, RRID:AB_477593), rabbit anti-alpha tubulin 1:1000 for WB (Abcam Cat# ab18251, RRID:AB_2210057), rabbit anti-AP4E1 1:1000 for WB (a gift from Margaret Robinson, University of Cambridge; Hirst et al., 1999), rabbit anti-ATG9A 1:1000 for WB and 1:100 for IF (clone EPR2450(2), Abcam Cat# ab108338, RRID:AB_10863880), guinea pig anti-beta tubulin III (TUJ1) 1:500 for IF (Synaptic Systems Cat# 302 304, RRID:AB_10805138), mouse anti-beta tubulin III (TUJ1) 1:800 for IF (clone SDL.3D10, Sigma-Aldrich Cat# T8660, RRID:AB_477590), rabbit anti-DAGLB 1:1000 for WB and 1:800 for IF (Abcam Cat# ab191159), chicken anti-GFP 1:500 for IF (Abcam Cat# ab13970, RRID:AB_300798), mouse anti-Golgin 97 (GOLGA1) 1:500 for IF (Abcam Cat# ab169287), mouse anti-HA 1:500 for IF (clone 16B12, BioLegend Cat# 901501, RRID:AB_2565006), mouse anti-Neurofilament (SMI312) 1:1000 for IF (BioLegend Cat# 837904, RRID:AB_2566782) and sheep anti-TGN46 1:200 for IF (Bio-Rad Cat# AHP500, RRID:AB_324049). Secondary antibodies used for WB were: Horseradish peroxidase (HRP)-conjugated goat anti-mouse IgG (Cat# AP308P, RRID:AB_11215796) and goat anti-rabbit IgG (Cat# AP307P, RRID:AB_11212848), purchased from Sigma-Aldrich and used at 1:5000, and near-infrared fluorescent-labelled secondary antibodies IRDye 680LT donkey anti-mouse IgG (IR680LT, Cat# 926-68022, RRID:AB_10715072) and IRDye 800CW donkey anti-rabbit IgG (IR800CW, Cat# 926-32213, RRID:AB_621848), purchased from LI-COR Biosciences and used at 1:10000. Fluorescently labelled secondary antibodies used in this study were Alexa Fluor Plus 488-labelled goat anti-chicken IgY (Cat# A32931, RRID:AB_2762843), Alexa Fluor 488-labelled goat anti-guinea pig IgG (Cat# A-11073, RRID:AB_2534117), Alexa Fluor 488-labelled goat anti-mouse IgG (Cat# A-11029, RRID:AB_2534088), Alexa Fluor 594-labelled goat anti-mouse IgG (Cat# A11005, RRID:AB_2534073), Alexa-Fluor Plus 680-labelled donkey anti-mouse IgG (Cat# A32788, RRID:AB_2762831), Alexa Fluor 568-labelled donkey anti-rabbit IgG (Cat# A10042, RRID:AB_2534017), Alexa Fluor 647-labelled goat anti-rabbit IgG (Cat# A21245, RRID:AB_2535813), and Alexa Fluor 680-labelled donkey anti-sheep IgG (Cat# A-21102, RRID:AB_2535755), all purchased from Thermo Fisher Scientific and used at 1:500.

Validation

The specificity of antibodies against the key protein targets of this study (human DAGLB & ATG9A) was confirmed in-house using siRNA-mediated knockdown in HeLa cells, by Western blotting (loss of band of expected size) and immunofluorescence microscopy (loss of fluorescent signal). The anti-ATG9A antibody is KO-validated by the manufacturer (Abcam). Fluorescently-labelled secondary

antibodies were confirmed to be specific through no primary antibody controls. The other primary antibodies, used as cellular markers, were not validated.

Eukaryotic cell lines

Policy information about cell lines

Cell line source(s)	HeLa M cells were a gift from Paul Lehner, University of Cambridge (Tiwari et al., 1987). Other cell lines used in this study were made in our lab and reported in previous publications: AP4B1 knockout HeLa (clone x2A3); wild-type AP4B1-rescued AP4B1 knockout HeLa (both described in Frazier et al., 2016); AP4E1 knockout HeLa (clone x6C3); CRISPR-Cas9 AP4B1-depleted SH-SY5Y, CRISPR-Cas9 AP4E1-depleted SH-SY5Y, and their Cas9-expressing parental cells (mixed populations); wild-type HeLa and AP4B1 knockout HeLa stably expressing RUSC2-GFP; AP4E1 knockout HeLa stably expressing GFP-RUSC2; endogenously-tagged HeLa SERINC1-Clover (clone A3) and HeLa SERINC3-Clover (clone B6) (all described in Davies et al., 2018). For HA-DAGLA and HA-DAGLB localisation experiments, a previously described HeLa cell line stably expressing GFP-tagged RUSC2 (clone 3; Davies et al., 2018) was re-single cell cloned for uniform expression to give HeLa GFP-RUSC2 clone D4. iPSCs from individuals with AP4B1-associated SPG47 and their sex-matched parents (heterozygous carriers, clinically unaffected) were generated previously in our lab (Teinert et al., 2019).
Authentication	Cell lines were not authenticated in the course of this study.
Mycoplasma contamination	All cell lines were routinely tested negative for the presence of mycoplasma contamination using DAPI to stain DNA and a PCR-based mycoplasma test (PanReac AppliChem ITW Reagents Cat# A3744).
Commonly misidentified lines (See ICLAC register)	No commonly misidentified cell lines were used.

Animals and other organisms

Policy information about studies involving animals; ARRIVE guidelines recommended for reporting animal research

Laboratory animals	Ap4e1 knockout mice (C57Bl/6N-Ap4e1[tm1b{KOMP}Wtsi]) were obtained via the European Mouse Mutant Archive (EMMA; strain EM:09451) from Helmholtz Zentrum München Deutsches Forschungszentrum für Gesundheit und Umwelt (GmbH). The brain samples used in this study were taken at 6 months (range 178-197 days). The WT group consisted of 4 females and 1 male; the KO group consisted of 3 females and 2 males. Mice were housed at an ambient temperature of 19-23 °C, recorded daily, with a humidity of 55% (+/- 10%). The light/dark cycle was 7 am (lights on) to 7 pm (lights off), with dawn and dusk periods, and animals had free access to standard animal food chow (SAFE® R105 diet from SAFE® Complete Care Competence) and water.
Wild animals	None
Field-collected samples	None
Ethics oversight	All animal procedures were conducted at the University of Cambridge, in accordance with the UK Animals (Scientific Procedures) Act of 1986 and under the authority of the UK Home Office Project Licence PPL 70/8339 held by Prof. Margaret Robinson, and were approved by the UK Home Office and the University of Cambridge Animal Welfare and Ethical Review Committee.

Note that full information on the approval of the study protocol must also be provided in the manuscript.
